# *Aegilops tauschii* genome assembly v6.0 with improved sequence contiguity differentiates assembly errors from genuine differences with the D subgenome of Chinese Spring wheat assembly IWGSC RefSeq v2.1

**DOI:** 10.1093/g3journal/jkaf042

**Published:** 2025-03-07

**Authors:** Rukmangada Maratikyathanahalli Srikanta, Le Wang, Tingting Zhu, Karin R Deal, Naxin Huo, Yong Q Gu, Patrick E McGuire, Jan Dvorak, Ming-Cheng Luo

**Affiliations:** Department of Plant Sciences, University of California, Davis, Davis, CA 95616, USA; Department of Plant Sciences, University of California, Davis, Davis, CA 95616, USA; Department of Plant Sciences, University of California, Davis, Davis, CA 95616, USA; Department of Plant Sciences, University of California, Davis, Davis, CA 95616, USA; Crop Improvement and Genetics Research Unit, USDA-ARS, Albany, CA 94710, USA; Crop Improvement and Genetics Research Unit, USDA-ARS, Albany, CA 94710, USA; Department of Plant Sciences, University of California, Davis, Davis, CA 95616, USA; Department of Plant Sciences, University of California, Davis, Davis, CA 95616, USA; Department of Plant Sciences, University of California, Davis, Davis, CA 95616, USA

**Keywords:** wheat, HiFi sequencing, Bionano genome map, hybrid scaffolding, assembly errors, orthologous genes, paralogous genes, collinear

## Abstract

*Aegilops tauschii* is the donor of the D subgenome of hexaploid wheat and a valuable genetic resource for wheat improvement. Several reference-quality genome sequences have been reported for *A. tauschii* accession AL8/78. A new genome sequence assembly (Aet v6.0) built from long Pacific Biosciences HiFi reads and employing an optical genome map constructed with a new technology is reported here for this accession. The N50 contig length of 31.81 Mb greatly exceeded that of the previous AL8/78 genome sequence assembly (Aet v5.0). Of 1,254 super-scaffolds, 92, comprising 98% of the total super-scaffold length, were anchored on a high-resolution genetic map, and pseudomolecules were assembled. The number of gaps in the pseudomolecules was reduced from 52,910 in Aet v5.0 to 351 in Aet v6.0. Gene models were transferred from the Aet v5.0 assembly into the Aet v6.0 assembly. A total of 40,447 putative orthologous gene pairs were identified between the Aet v6.0 and Chinese Spring wheat IWGSC RefSer v2.1 D-subgenome pseudomolecules. Orthologous gene pairs were used to compare the structure of the *A. tauschii* and wheat D-subgenome pseudomolecules. A total of 223 structural differences were identified. They included 44 large differences in sequence orientation and 25 differences in sequence location. A technique for discriminating between assembly errors and real structural variation between closely related genomes is suggested.

## Introduction

*Aegilops tauschii* (2*n* = 2*x* = 14, genomes DD) is the donor of the D subgenome of hexaploid wheat (*Triticum aestivum*, 2*n* = 6*x* = 42, subgenomes AABBDD) (Kihara 1944; McFadden and Sears 1946). The species consists of 2 major phylogenetic lineages, designated as 1 and 2 (Wang *et al*. 2013), and 1 minor lineage, designated as 3 (Gaurav *et al*. 2021). The bulk of the wheat D subgenome was contributed by lineage 2 in northwestern Caspian Iran (Wang *et al*. 2013) and a small portion of it was introgressed from lineage 3 (Gaurav *et al*. 2021) and lineage 1 (Wang *et al*. 2013; Cavalet-Giorsa *et al*. 2024).

*Aegilops tauschii* is distributed from East Turkey to China and West Pakistan (Van Slageren 1994) and is adapted to a diversity of environments across its wide geographic range. The incorporation of the *A. tauschii* genome into hexaploid wheat increased wheat stress tolerance and environmental adaptability (Dubcovsky and Dvorak 2007). *Aegilops tauschii* remains an important genetic resource for wheat breeding, primarily for disease resistance (Gill *et al*. 1986; Raupp *et al*. 1993; Cox *et al*. 1994; Periyannan *et al*. 2013; Gaurav *et al*. 2021; Kou *et al*. 2023; Cavalet-Giorsa *et al*. 2024). Synthetic wheat produced by hybridization of tetraploid wheat with *A. tauschii* is an important source of new variation for broadening genetic diversity of the wheat D subgenome (Yang *et al*. 2009; Ogbonnaya *et al*. 2013; Li *et al*. 2014; Das *et al*. 2016; Cox *et al*. 2017; Rosyara *et al*. 2019; Wan *et al*. 2023).

The overall structure of *A. tauschii* chromosomes approximates the 7 chromosomes of the hypothetical progenitor of the tribe Triticeae (Luo *et al*. 2009). The *A. tauschii* genome is, therefore, a logical reference in analyses of chromosome structure and gene orthology in the tribe and the grass family (Luo *et al*. 2017; Sun *et al*. 2017; Dvorak *et al*. 2018).

For these basic and applied reasons, it is important that the genome sequence of *A. tauschii* keeps pace with advances in genome sequencing technology. That is particularly true as the *A. tauschii* genome is large (>4.2 Gb) and contains as much as 84.4% of repeated nucleotide sequences (Luo *et al*. 2017). As most of the *A. tauschii* repeated nucleotide sequences evolved recently (Dai *et al*. 2018), the *A. tauschii* genome contains many homogeneous repeated sequence families (Luo *et al*. 2017; Dai *et al*. 2018). Due to the large size of the genome and excessive homogeneity of repeated sequence families, the assembly of the *A. tauschii* genome sequence has been a challenging endeavor.

Two reference-quality genome sequences have been reported for *A. tauschii* acc. AL8/78 (lineage 2, Armenia) (Luo *et al*. 2017; Zhao *et al*. 2017). The genome sequence reported by Zhao *et al*. (2017) was assembled on a contractual basis by NRGene Inc. from short, whole-genome shotgun (WGS) Illumina reads. A sequence reported by Luo *et al*. (2017) was assembled by a combination of approaches. First, a physical map was built from bacterial artificial chromosomes (BACs) (Luo *et al*. 2013). The map was used to generate a BAC-by-BAC genome sequence. This sequence was merged with: (1) a sequence built by NRGene Inc. on a separate contractual basis from WGS Illumina reads and (2) mega-reads built from Pacific Biosciences (PacBio) reads by Zimin *et al*. (2017). The resulting assembly was validated with 3 genome-wide Bionano Genomics (henceforth Bionano) optical maps. This was assembly Aet v4.0 (Luo *et al*. 2017). The 7 Aet v4.0 pseudomolecules were 4,025 Mb long, while an additional 200 Mb of scaffolds were not assigned to pseudomolecules. Aet v4.0 was later improved by the deployment of additional Bionano optical maps, community annotation, and annotation of small RNAs yielding assembly Aet v5.0 (Wang *et al*. 2021).

Further improvements of the *A. tauschii* acc. AL8/78 reference genome sequence, resulting in assembly Aet v6.0, are reported here. This assembly was produced with the PacBio HiFi single-molecule sequencing technology and an optical map constructed with the Bionano direct-label-and-stain (DLS) chemistry.

The first-generation Bionano optical mapping chemistry utilized a single-strand restriction endonuclease to nick DNA, which was followed by enzymatic repairing and labeling of DNA nicks (Hastie *et al*. 2013; Luo *et al*. 2017). This chemistry was known as the nick, label, repair, and stain (NLRS) chemistry. Chance clustering of nick sites produced fragile regions in DNA molecules that were prone to breakage during sample preparation, thus limiting the length of optical contigs that could be assembled. In contrast, DLS chemistry labels DLE-1 restriction enzyme recognition sites directly without nicking DNA. This chemistry does not produce these fragile regions, and therefore longer optical contigs can be produced with it.

Here, DLE-1 optical mapping was combined with the long-read, single-molecule PacBio HiFi sequencing technology to produce the Aet v6.0 assembly. This hybrid approach produced scaffolds and super-scaffolds (SSs) with greater contiguity and improved structure compared to those of the prior Aet v5.0 assembly. These scaffolds and SSs were anchored on a high-resolution genetic map of *A. tauschii* (Luo *et al*. 2013), and Aet v6.0 pseudomolecules were constructed. Genes annotated in Aet v5.0 were transferred into the Aet v6.0 assembly.

To visualize structural differences between the Aet v5.0 and Aet v6.0 pseudomolecules, the pseudomolecules of the 2 assemblies were compared with a dot plot and also by aligning the pseudomolecules of the 2 assemblies on the DLE-1 optical genome map. As *A. tauschii* is the progenitor of the D subgenome of bread wheat, gene orthology between Aet v6.0 and the D subgenome of the reference genome sequence for Chinese Spring (CS) wheat, IWGSC RefSeq v2.1 (Zhu *et al*. 2021), was also assessed. Cultivar CS has served as a reference for wheat genetics since the 1950s and its genome sequence is an important reference for wheat genetics and genomics. Collinearity of orthologous gene pairs along pseudomolecules was used to detect structural differences between the 2 assemblies. Finally, a technique was described and evaluated for differentiating real structural differences from errors in assembly between the *A. tauschii* acc.AL8/78 genome and the CS wheat D subgenome.

## Methods

### Pacific Biosciences HiFi sequencing

High-molecular-weight (HMW) DNA was isolated from young leaves of *A. tauschii* acc. AL8/78 as described earlier (Dvorak *et al*. 1988). DNA was sheared to fragments between 15 and 20 kb using the Megaruptor 2 (Diagenode, Denville, NJ, USA). HiFi libraries were prepared using the SMRTbell Express Template Prep Kit 2.0 and cleaned with the Enzyme Clean up Kit (Pacific Biosciences, Menlo Park, CA, USA). The libraries were size selected utilizing BluePippin (Sage Sciences, Beverly, MA, USA). HiFi SMRTbell templates longer than 15 kb were collected and cleaned with AMPure PB beads (Pacific Biosciences). Concentration and final size distribution of the libraries were evaluated using a Qubit 1× dsDNA HS Assay Kit (Thermo Fisher, Waltham, MA, USA) and Femto Pulse System (Agilent, Santa Clara, CA, USA), respectively. HiFi libraries with approximate insert sizes of 15 kb were independently sequenced on 2 SMRT cells of a Pacific Biosciences Sequel II instrument (Pacific Biosciences) at the Vincent J. Coates Genomics Sequencing Lab, University of California, Berkeley, and assembled using Hifiasm v0.16.1-r374 with parameters of “-l 0 -f 39” (Cheng *et al*. 2021) to generate a primary assembly contig graph.

### Construction of the DLE-1 optical map

To construct DLE-1 optical map of *A. tauschii* acc. AL8/78, HMW-DNA was isolated from young leaves following the Bionano Prep Plant Tissue DNA Isolation Base Protocol (Bionano Genomics, San Diego, CA, USA). DNA was labeled with the DLE-1 restriction enzyme and stained according to instructions in the Bionano Prep Direct Label and Stain Kit (Bionano Genomics). Labeled molecules were scanned with the Saphyr system (Bionano Genomics). The consensus optical map was de novo assembled with the Assembler tool in the Bionano Solve v3.2 package using significance cutoffs of *P* < 1 × 10^−10^ to generate a draft consensus map (CMAP), *P* < 1 × 10^−11^ for draft CMAP extension, and *P* < 1 × 10^−15^ for final merging of the draft CMAP while choosing the “nonhaplotype”, “noES”, and “noCut” options.

### Scaffolding

Scaffolds were assembled with the hybrid scaffold pipeline in the Bionano Solve v3.3 package (Bionano Genomics). The scaffolding process consisted of 5 major steps. (1) In silico DLE-1 restriction maps were generated for the PacBio HiFi sequence assembly. (2) Problematic regions were manually analyzed by altering contig assembly stringency, and by aligning the in silico maps (CMAPs) against the DLE-1 optical map. (3) Hybrid scaffolds consisting of HiFi sequence contigs and DLE-1 optical contigs aligned on each other were generated. If a DLE-1 contig aligned to 2 or more HiFi contigs, the HiFi contigs were oriented and linked. The sizes of gaps between sequence contigs were estimated based on the DLE-1 map. (4) Sequence CMAPs and hybrid scaffolds were aligned. (5) AGP and FASTA files were generated for the hybrid SSs. Conflicts between sequence and optical contigs were manually examined and resolved, which produced the final set of SSs.

### Anchoring of super scaffolds on a genetic map

SSs generated with the hybrid scaffolding pipeline were aligned on a high-density SNP-based genetic map built from marker segregation in a biparental AL8/78 × AS75 F_2_ mapping population (Luo *et al*. 2013). The map consisted of 7,185 SNP marker loci (Luo *et al*. 2013). The order and orientation of SSs along each chromosome were determined using a basic local alignment search tool (BLAST). The SSs were aligned using 99% identity with the *e*-value >10^−5^. All cases in which 2 SNP markers that were not physically close to each other, but were present in the same SS, or in which SNP markers were located in more than 1 SS, were manually scrutinized.

### Pseudomolecule construction, validation, and analysis

SSs ordered on the genetic map were joined with ‘N’ characters while strand orientation of neighboring SSs was considered. This produced Aet v6.0 pseudomolecules.

The following strategy was used to validate and analyze the Aet v6.0 pseudomolecules. Each Aet v6.0 pseudomolecule was in silico digested (Bionano Access Software) to create its consensus DLE-1 restriction map (CMAP) and was then anchored back to the SS CMAP using the RefAligner tool (Bionano Access Software). Also digested in silico were Aet v5.0 pseudomolecules (Wang *et al*. 2021), and CMAPs were created for them as well. The Aet v5.0 pseudomolecule CMAPs and Aet v6.0 pseudomolecule CMAPs were aligned. The ordering and orientation of alignments were manually inspected to identify problem regions. The Nucmer alignment tool (Marcais *et al*. 2018) was used to verify the Aet v6.0 and Aet v5.0 assemblies. The output-coordinated files obtained from Nucmer were used to generate a dot plot (Marcais *et al*. 2018) for the visualization of discrepancies between the 2 assemblies, such as incorrectly placed sequence segments, duplicated or missing segments, and incorrectly oriented sequence segments.

### Gene model transfer from Aet v5.0 to Aet v6.0

Genes models were transferred from Aet v5.0 to Aet v6.0 with the Liftoff program (Shumate and Salzberg 2021) using default parameters; liftoff –g.gff3 -o.gff3 -u.txt -copies -chroms.fasta.fa and the Aet v5.0 gene models. Based on their coordinates, the transcript sequences were extracted with the gffread program (Pertea and Pertea 2020), and putative open reading frames of transcripts and corresponding protein sequences were determined with Transdecoder v5.7.0 (https://github.com/TransDecoder/TransDecoder/releases) using the following parameters: -m 30 --retain_pfam_hits –retain_blastp_hits. The classification of transferred genes as high-confidence (HC) and low-confidence (LC) genes [International Wheat Genome Sequencing Consortium (IWGSC) 2014] assigned to the genes in the Aet 5.0 assembly was retained. The predicted protein sequences were mapped against PfamAB.hmm (Mistry *et al*. 2021) and filtered based on homology modeling, the number of PFAM domains, the number of significant BLAST hits, and the total length of coding sequence (CDS) with start and stop codon. The completeness of gene transfer was evaluated by searches against 4,956 BUSCO genes using BUSCO software (BUSCO 4.1.4) (Manni *et al*. 2021). For each BUSCO gene absent from Aet v6.0, it was determined if it was also absent in Aet v5.0 (Wang *et al*. 2021).

### Orthologous relationships between Aet v6.0 genes and those of the Chinese Spring D subgenome

A BLAST (Camacho *et al*. 2009) homology search with the parameter setting -max_target_seqs 3 was performed with the Aet v6.0 genes used as queries and the CS IWGSC RefSeq v2.1 D-subgenome genes (Zhu *et al*. 2021) as targets. The following data were recorded: top 3 hits including gene name, locations in the CS v2.1 D-subgenome pseudomolecule registry in base pairs (bp), sequence identity in % of bp, CDS length, and the length of alignment of the query/target genes.

The strategy for discovering orthologous genes was derived from the concept of “conserved gene neighborhood” (CGN). CGN was defined as genomic segments containing orthologous genes in a similar collinear order (Kuzniar *et al*. 2008). This definition was turned around for defining orthologous genes: “A query gene and top BLAST hit gene in a collinear order in a CGN are orthologous.” This definition and its use are illustrated by the following example. Consider the ascending sequence of query genes A B C D E F G along a segment of an Aet v6.0 pseudomolecule. Assume that the top BLAST gene hits along a corresponding segment of a D-subgenome pseudomolecule were in the following ascending order: A B D E F C G. Genes A B D E F G were in a collinear order in the CGN along the segment in Aet v6.0 and CS D subgenome and each of them should be a member of an orthologous gene pair. Genes C were in noncollinear locations and most likely were a product of gene duplication/deletion. Hence, genes C should be a paralogous gene pair.

However, it cannot be excluded for any of the A B D E F G collinear gene pairs that they have not been subjected to a tandem duplication in *A. tauschii* followed by gene deletions. For example, a tandem duplication of gene E in *A. tauschii* prior to the origin of hexaploid wheat would produce an in-paralogous tandem gene duplication E_1_ E_2_. If E_1_ was deleted from the *A. tauschii* chromosome while E_2_ was deleted from the D-subgenome chromosome, E genes would be out-paralogues E_1_ and E_2_ in the 2 pseudomolecules, despite their apparent collinearity in the CGN. A reverse scenario could be visualized for gene C. If gene C was moved to a new position by transposition without a gene duplication, the 2 C genes would be orthologues, despite the CGN being perturbed. For both reasons, absolute orthologous/paralogous gene relationships are difficult to prove. As a recognition of that fact, we will refer to the gene pairs identified by our method as putative orthologous and putative paralogous gene pairs.

A complicating situation was when neighboring Aet v6.0 query genes were duplicated and both hit the same target gene. In such cases, the percent of gene sequence identity and length of query–target alignment were used to infer which of the 2 query genes was a putative orthologue and which was a putative paralogue. Threshold sequence identity of 97.0% was chosen based on mean sequence identity *μ* = 99.4% between 40,448 putative orthologous gene pairs (Supplementary Table 1), a standard deviation *σ* = 1.65%, and the level of significance *α* = 0.01. An orthology/paralogy inference was not made for duplicated query genes below the threshold.

## Results and discussion

### Assembly

Two Sequel II SMRT cells were sequenced with third-generation PacBio HiFi sequencing technology. A total of 8.05 million high-quality HiFi reads with an average read length of 14.9 kb were obtained and assembled into 1,367 contigs with a maximum length of 131 Mb and an N50 contig length of 31.8 Mb. A sequencing depth of 20× or more is needed for a genome sequence assembly from HiFi reads (Zhang *et al*. 2023). As the total depth of our HiFi reads was 29.3× genome equivalents, no polishing of the 1,367 contigs was pursued, as would otherwise be needed with a lower depth of HiFi reads (Zhang *et al*. 2023).

To deploy the Bionano hybrid scaffold pipeline in the assembly, a Bionano DLE-1 optical map for *A. tauschii* acc. AL8/78 was constructed. A total of 3,670,984 molecules (851.02 Gb) were used to assemble the Bionano optical genome map. A total of 254 contigs of a total length of 4,060.33 Mb and N50 of 45.11 Mb were obtained (Table 1). This contig N50 greatly surpassed the contig N50 of 2.17 Mb obtained with the NLRS chemistry (Luo *et al*. 2017). The total length of the optical map was close to the genome size reported for AL8/78, 4.22 Gb, based on the total sequence length of the Aet v4.0 assembly (Luo *et al*. 2017).

**Table 1.** Metrics for the genome-wide DLE-1 optical map of *Aegilops tauschii* acc. AL8/78.

Metric	
Molecules (no.)	3,670,984
Total length of molecules (Gb)	851.02
Average length (Kb)	231.82
Molecule N50 (Kb)	224.79
Label density (per 100 Kb)	12.77
Coverage of the reference genome (X)	208.59
Contigs (no.)	254
Map total length (Mb)	4,060.33
Mean contig length (Mb)	15.98
Contig N50 (Mb)	45.11
Maximum contig length (Mb)	153.28

Using the Bionano hybrid scaffold pipeline, sequence scaffolds and DLE-1 optical contigs were aligned and neighboring sequence scaffolds were linked. Conflicts were manually resolved. Ultimately, a final set of 1,254 SSs/contigs was produced. Fifty-five contigs were eliminated (52 were mitochondrial DNA contaminations, and 3 were <200 bp long), leaving 1,199 SS/contigs for the construction of pseudomolecules.

To generate pseudomolecules, SSs/contigs were anchored on a high-density, SNP-based genetic map (Luo *et al*. 2013). Of the 1,199 SSs/contigs, 92 SSs with a combined length of 4,055 Mb, representing 97.99% of the total SSs/contig length, were anchored. The 92 anchored SSs produced 7 Aet v6.0 pseudomolecules (Table 2). The remaining 1,107 SSs/contigs of a combined total length of 67.68 Mb and representing 1.64% of the total assembly length could not be anchored and were excluded from pseudomolecule construction (Table 2).

**Table 2.** Anchoring of SSs with SNP markers on the genetic map, thereby producing Aet v6.0 pseudomolecules.

Pseudomolecule	SSs (no.)	SNPs (no.)	Length (bp)	Ns (no.)
Chr1	11	967	507,515,187	188,860
Chr2	12	1,299	654,279,071	57,881
Chr3	16	1,097	629,928,798	213,896
Chr4	15	786	528,822,301	104,851
Chr5	10	1,004	584,392,136	236,951
Chr6	9	747	497,522,035	233,166
Chr7	19	1,102	652,241,641	335,886
Total	92	7,002	4,054,701,169	1,371,491

The number of gaps and their combined total length was significantly reduced in the Aet v6.0 pseudomolecules compared to Aet v5.0 pseudomolecules; 351 gaps in Aet 6.0 vs 52,910 in Aet 5.0 (Table 3). Ninety-one of the 351 gaps were of an unknown length. For those, 100 Ns were inserted into the gap to link adjacent SSs. The remaining 260 gaps were of a known length, which ranged from 23 to 65,714 bp and were filled with corresponding numbers of Ns. In total, there were 1.37 million Ns in the pseudomolecules (Table 2).

**Table 3.** Comparison of the Aet v5.0 and Aet v6.0 pseudomolecules.

Pseudomolecule	Assembly	Total length (bp)	Gap length (bp)	Effective length (bp)	Gaps (no.)
Chr1	Aet v5.0	501,967,303	8,406,331	493,560,972	6,647
	Aet v6.0	507,515,187	188,860	507,326,327	39
Chr2	Aet v5.0	650,458,083	8,558,830	641,899,253	8,139
	Aet v6.0	654,279,071	57,881	654,221,190	36
Chr3	Aet v5.0	627,456,150	9,202,775	618,253,375	9,437
	Aet v6.0	629,928,798	213,896	629,714,902	63
Chr4	Aet v5.0	525,206,139	8,086,205	517,119,934	6,175
	Aet v6.0	528,822,301	104,851	528,717,450	48
Chr5	Aet v5.0	576,238,907	12,629,354	563,609,553	7,615
	Aet v6.0	584,392,136	236,951	584,155,185	51
Chr6	Aet v5.0	495,363,004	7,581,178	487,781,826	6,199
	Aet v6.0	497,522,035	233,166	497,288,869	38
Chr7	Aet v5.0	644,841,383	14,867,162	629,974,221	8,698
	Aet v6.0	652,241,641	335,886	651,905,755	76
v6.0-v5.0		33,170,200	−67,960,344	101,130,544	−52,559
Probability (*t*-test)		*P* = 0.23	*P* = 0.0001	*P* = 0.005	*P* = 0.0001

### Pseudomolecule validation

**Fig. 1.**
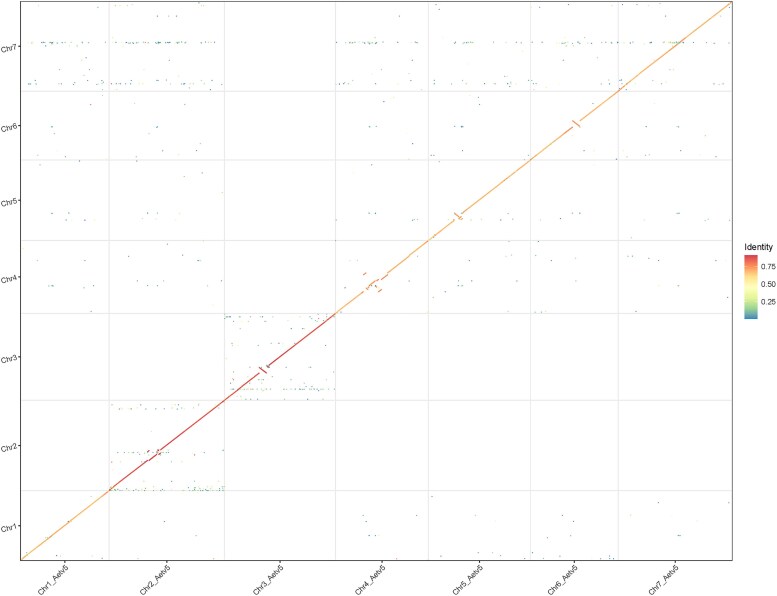
Dot-plot between Aet v5.0 and Aet v6.0 pseudomolecules. The Aet 5.0 pseudomolecules are on the vertical axis and Aet v6.0 pseudomolecules are on the horizontal axis. Note differences in orientation and placement of scaffolds within pseudomolecules for Chr2, Chr3, Chr4, Chr5, and Chr6. Sequence identity scale is on the right.

The Aet v6.0 pseudomolecules were compared with the Aet v5.0 pseudomolecules by a genome-wide dot-plot (Fig. 1). The dot-plot hinted at structural differences between the 2 assemblies within 6 of the 7 pseudomolecules, but no differences among pseudomolecules were apparent (Fig. 1). The differences primarily consisted of the orientation and placement of segments within pseudomolecules.

**Fig. 2.**
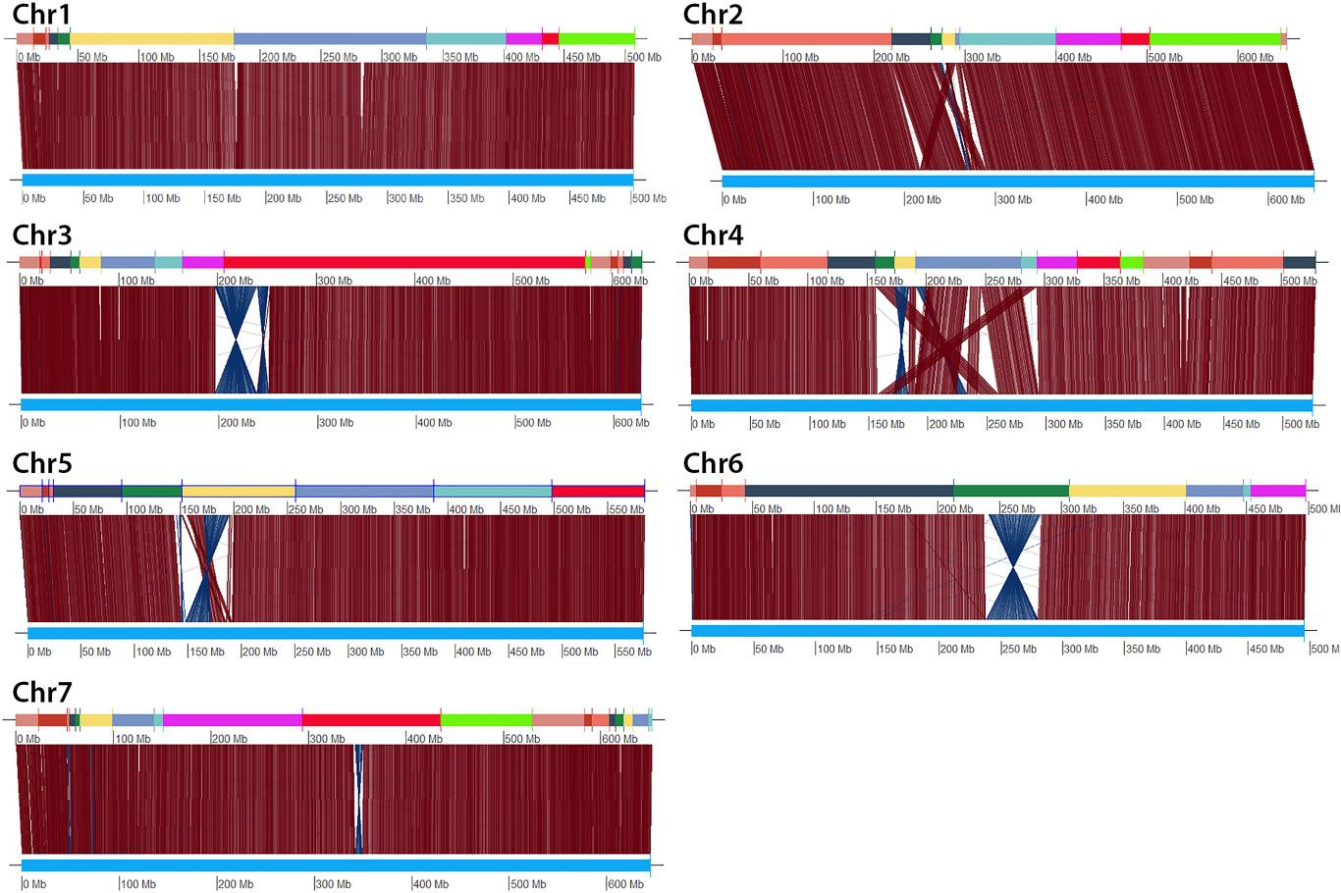
Alignments of corresponding DLE-1 restriction sites between homologous Aet v5.0 and Aet v6.0 pseudomolecules. In each alignment, the sequence of Aet v6.0 SSs is at the top and the Aet v5.0 pseudomolecule is at the bottom. The vertical and/or diagonal lines between these 2 tracks connect the locations of corresponding DLE-1 restriction sites.

To visualize differences between homologous Aet v5.0 and Aet v6.0 pseudomolecules in greater detail, in silico DLE-1 digests of homologous pseudomolecules were aligned (Fig. 2). The alignments revealed differences between all 7 pseudomolecule pairs. Small sequence insertions/deletions were apparent in all pseudomolecule pairs, and differences in the orientation or ordering of sequence segments were apparent in the centromeric regions of 6 of the 7 pseudomolecule pairs (Fig. 2).

**Fig. 3.**
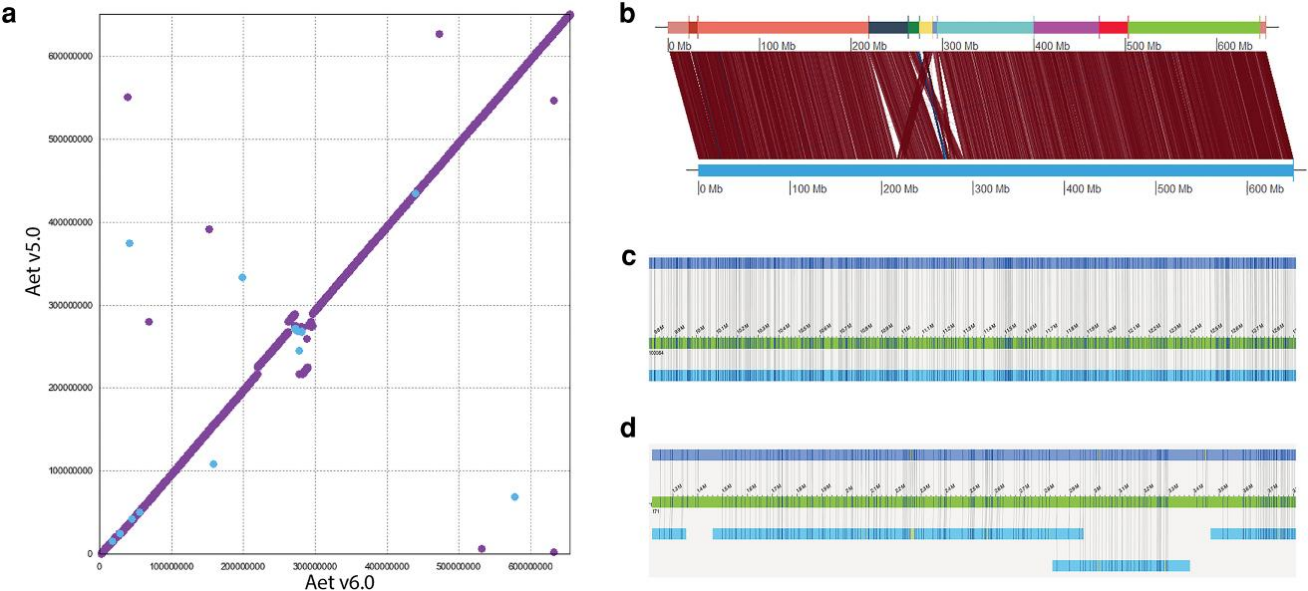
Veracity of differences between Chr2 pseudomolecules in Aet v5.0 and Aet v6.0. a) Dot-plot showing differences in scaffold ordering and orientation between the Aet v5.0 and Aet v6.0 pseudomolecules for Chr2. b) As in Fig. 2, the top track is the sequence of 12 Aet v6.0 SSs (arbitrarily colored) and the bottom track is the Aet v5.0 pseudomolecule. The vertical and/or diagonal lines between these 2 tracks connect the locations of corresponding DLE-1 restriction sites. The alignment of DLE-1 sites confirmed the differences in scaffold ordering and orientation shown by the dot plot a). c, d) In each image, the top track is a representation of the relevant HiFi SSs of Aet v6.0; the middle track with the base-pair register is the HiFi-DLE-1 hybrid scaffold, and the bottom track, in c), or bottom 2 tracks, in d), are the DLE-1 optical scaffolds. Two regions in Aet v6.0 showed reverse orientation compared to Aet v5.0: SC100084 (the fifth SS from the left in the top track in b) and SC171 (the sixth SS from the left in the top track in b)]. c) The SC100084 region, spanning 9,765,966–12,911,409 bp, is oriented in b) in the opposite direction in Aet v5.0 compared to its orientation in Aet v6.0. However, the HiFi-DLS hybrid scaffolds (middle track) are oriented in the same direction as those in SC100084 (top track) and on the optical map (lower track), indicating that the orientation of the region in Aet v6.0 is correct. d) The region spanning 1,504,058–1,509,984 bp in scaffold SC171 (top track) is collinear with HiFi-DLE-1 hybrid scaffold (middle track) and the DLS optical map (lower track), indicating that the orientation of the region in Aet 6.0 is correct.

The following approach, illustrated with the alignments of pseudomolecules for Chr2 (Fig. 3), was used to determine which of the 2 pseudomolecules was correct. Segments in which Aet v5.0 and Aet v6.0 pseudomolecules differed were aligned on the DLE-1 optical genome map (Fig. 3). A pseudomolecule showing the same structure as the optical map was concluded to be correct. This approach indicated that the differences between the Aet v6.0 and Aet v5.0 pseudomolecules were likely errors in the Aet v5.0 assembly.

### Gene model transfer from the Aet v5.0 assembly to the Aet v6.0 assembly

A total of 68,383 genes, 32,712 HC and 35,671 LC genes, were transferred from Aet 5.0 into Aet 6.0 (Table 4). There were 173 more HC genes and 232 more LC genes in Aet v6.0 pseudomolecules compared to the Aet v5.0 assembly. While some of these additional genes are genes that were on unassigned scaffolds in Aet v5.0, some may be errors in the gene transfer, which may have generated spurious duplicated genes. A total of 1,920 artifactual duplicated genes in addition to the gene models present in Aet v5.0 were encountered in an earlier draft of gene transfer to Aet v6.0, most of them having the same start and end bp coordinates. Most of these genes were removed, but 115 of such gene pairs that had the same start bp coordinates, but differed in the ending nucleotide and when used as BLAST queries against wheat D subgenome genes IWGSC RefSeq v2.1 hit different target genes were left in the Aet v6.0 gene database (Supplementary Table 1). These spurious duplicated genes were labeled with an exclamation mark in column D of Supplementary Table 1.

**Table 4.** The numbers of HC and LC genes in the 7 *A. tauschii* Aet v6.0 pseudomolecules, the numbers of putative orthologous gene pairs, the numbers of putative paralogues gene pairs, gene pairs for which orthologous/paralogous relationship could not be estimated (uncertain), and numbers of query genes for which target genes were undetected by BLAST in the CS IWGSC RefSeq v2.1 pseudomolecules.

Chr.	All		Ortholog.		Paralog.		Uncertain		Undetected	
	HC	LC	HC	LC	HC	LC	HC	LC	HC	LC
Chr1	4,165	4,790	3,589	1,524	428	2,434	95	162	53	670
Chr2	5,543	5,831	4,883	2,019	436	2,691	166	337	58	780
Chr3	5,172	5,400	4,495	1,896	417	2,442	170	318	90	744
Chr4	3,616	3,830	3,167	1,312	293	1,842	70	166	86	510
Chr5	5,219	5,277	4,677	1,804	323	2,404	151	333	68	736
Chr6	3,895	4,401	3,282	1,405	386	2,152	85	185	142	559
Chr7	5,102	6,144	4,405	1,989	466	2,918	154	274	77	963
Total	32,712	35,671	28,498	11,949	2,749	16,883	891	1,775	574	5,066
%	47.8	52.2	87.1	33.5	8.4	47.3	2.7	5.0	1.7	15.5

In addition to BLAST searches in Supplementary Table 1, the completeness of gene model transfer was assessed by searches for a set of 4,956 BUSCO genes in Aet v6.0 and Aet v5.0. A total of 4,584 (92.5%) BUSCO genes were found in the Aet v6.0 assembly and 4,592 (92.6%) were in the Aet v5.0 assembly. The close agreement in the detection of BUSCO genes in the 2 assemblies confirmed that gene model transfer from Aet v5.0 to Aet v6.0 was adequate. Nevertheless, de novo reannotation of the Aet v6.0 genome sequence using an approach analogous to that recently applied to the human genome (Varabyou *et al*. 2023) would be desirable.

### Putative orthologous genes in the Aet v6.0 and CS IWGSC RefSeq 2.1 D-subgenome assemblies

We considered it of interest to ask how much agreement was between Aet v6.0 and the most recent sequence assembly of the D subgenome of CS wheat, IWGSC RefSeq v2.1 (Zhu *et al*. 2021). We employed gene collinearity along pseudomolecules and BLAST searches to identify putative orthologous gene pairs between Aet v6.0 and CS D subgenome as detailed in *Methods*.

Briefly, each of the 68,383 Aet v6.0 genes (Table 4) was used as a query in BLAST searches against the CS v2.1 D-subgenome gene set. The locations of the top 3 CS target gene hits on D-subgenome pseudomolecules were recorded in a spreadsheet (Supplementary Table 1). Also recorded was percent sequence identity and the length of query/target alignment for each query and target gene pair. If query and top target genes were in a collinear ascending or descending order, they were classified as putative orthologues. If they were noncollinear, they were classified as putative paralogues. If the BLAST search failed to detect any target, such a query gene was classified as undetected in the CS D subgenome (Table 4). If juxtaposed query genes hit the same target gene and both showed sequence identity less or more than 97%, the orthologous/paralogous decision was not made (Table 4).

A total of 28,498 (87%) putative orthologous gene pairs were identified for the 32,712 Aet v6.0 HC genes, but only 11,949 (33.5%) of putative orthologous gene pairs were identified for the 35,671 LC genes (Supplementary Table 1, Table 4) (*P* < 0.001, paired *t*-test). A reverse trend was encountered for the putative paralogous gene pairs. A total of 16,883 (47.3%) paralogous gene pairs were encountered for the 35,671 LC Aet v6.0 genes but only 2,749 (8.4%) paralogous gene pairs were encountered for the 32,712 HC Aet v6.0 genes (*P* < 0.001, paired *t*-test) (Table 4). For 891 HC and 1,775 LC query genes, an orthologous/paralogous decision was not made (Table 4).

The number of putative orthologous gene pairs found here, 40,447, greatly exceeded 6,449 orthologous gene pairs previously estimated for the same *A. tauschii* accession and the same wheat cultivar (Sun *et al*. 2017). While the number of orthologues may be overestimated here, as pointed out in *Methods*, the difference between the 2 estimates is too large to be attributable only to an overestimation. There would have to be approximately 4 duplication/deletion events of the type described in *Methods* for every real orthologous gene pair. Fragmented genome sequences or a too-conservative algorithm used by Sun *et al*. (2017) may have likely caused this large difference.

The average sequence identity for 40,447 putative orthologous gene pairs (Supplementary Table 1) was 99.4% (*σ* = 1.65%). Of this, the average sequence identity for the HC orthologous genes was 99.6% (*σ* = 1.2%), and for the LC orthologous genes was 99.3% (*σ* = 1.3%). The average sequence identity between putative paralogous HC and LC genes was 93.3% (*σ* = 6.7%) and 95.2% (*σ* = 4.5%), respectively (Supplementary Table 1).

Collinear putative orthologues were used to identify differences in orientation and locations of sequence segments in Aet v6.0 and CS RefSeq v2.1 assemblies (Table 5). Differences in orientation were allocated into 3 classes based on the number of gene pairs involved (Table 5). Those involving 2 gene pairs were the most common. The second most common type was differences in segment orientation involving 4 or more gene pairs (Table 5).

**Table 5.** Structural differences between the Aet v6.0 and CS RefSeq v2.1 pseudomolecules.

Pseudomolecule	Difference in orientation			Difference in location	
	2 genes	3 genes	>3 genes	Intrachr.	Interchr.
Chr1	14	2	7	2	1
Chr2	31	3	5	4	0
Chr3	23	2	8	1	1
Chr4	7	4	8	4	2
Chr5	22	3	6	0	0
Chr6	23	1	4	4	2
Chr7	14	5	6	4	0
Total (no)	134	20	44	19	6

Differences in segment location were considered only if the segment involved 3 or more collinear orthologous gene pairs. Differences in segment location within a pseudomolecule were more common than those among pseudomolecules (*P* = 0.026, paired *t*-test) (Table 5).

### Application of BLAST sequence identity in identifying potential errors in an assembly

Collinearity analyses between Aet v6.0 and CS D-subgenome pseudomolecules revealed 6 interchromosomal location differences (labeled red in Supplementary Table 1) and 19 intrachromosomal location differences (labeled blue in Supplementary Table 1) of homologous segments (Supplementary Table 1, Table 5). If a location difference were an assembly error, the Aet and CS segments would be orthologous segments placed in a wrong place in one of the assemblies. If a location difference were real, the segments would likely be paralogous to each other because they were produced by segmental duplication. In the former case, collinear genes along a segment would be putative orthologues, but in the latter case, they would be putative paralogues. The average sequence identity of collinear gene pairs across 5 of the 6 pairs of segments located in different pseudomolecules ranged from 92.0% to 95.7% with a mean of 94.9% (Supplementary Table 1), which was similar to the average sequence identity for paralogous gene pairs. The average sequence identity was 100% across the sixth segment (genes AET4D6G0108200LC to AET4D6G0108300LC, Supplementary Tables 1). Based on the above reasoning this likely was the only assembly error among the 6 segments.

The same rationale was applied to the 19 intrachromosomal segment location differences. They too fell into 2 classes. The mean identity of collinear gene pairs across a segment ranged from 78.8% to 97.1% with a mean of 92.2% for 6 segments but ranged from 98.6% to 99.9%, with a mean of 99.5%, for the remaining 13 segments (*P* = 0.04, 2-tailed *t*-test with unequal variance). Based on the same reasoning as above, we suggest that the former 6 segments may have been real intrachromosomal translocations, but the latter 13 segment location differences were assembly errors.

We attempted to apply this approach to the 44 differences in the orientation of segments. The average sequence identity ranged from 100% to 93.7% across the 44 segments with a mean of 98.7% (*s* = 1.67%). The average sequence identity of collinear genes across a segment failed to show a clear bimodal density distribution, possibly due to the fact that segments differing in orientation (inversions) cannot be paralogous.

However, a comparison of the average sequence identity of collinear genes within a segment with that of flanking segments can potentially be used to discriminate between orientation errors in assembly from real inversions. Inversion polymorphism in a population is in a heterozygous state for a substantial length of time after its origin. During that time, heterozygosity acts as a recombination suppressor and precludes purifying selection from acting against mutations in genes in the segment, leading to faster divergence and lower sequence identity between collinear genes within the segment than genes flanking it.

Consider 2 segments differing by orientation between Aet v6.0 and CS D subgenome on pseudomolecules for Chr3. An inversion starting with *A. tauschii* gene AET3D6G0489500 (Supplementary Table 1) involved 4 collinear genes with an average identity of 93.6%. Sequence identity of 10 genes on the distal side of the segment was 98.9% (*P* = 0.05, *t*-test) and sequence identity on the proximal side of the segment was 99.0% (*P* = 0.009, *t*-test). Since the collinear genes in the inverted segment were more diverged than those in the flanking segments, this orientation difference was likely due to a real paracentric inversion. An opposite inference was made for the difference in segment orientation including 21 collinear genes starting with *A. tauschii* gene AET3D6G0502800 on pseudomolecule Chr3 (Supplementary Table 1). The average identity of the collinear genes was 96.9% and that of flanking 13 and 8 collinear genes was 99.4% (*P* = 0.06, *t*-test) and 97.8% (*P* = 0.59, *t*-test), respectively. In this case, the divergence of flanking collinear genes between Aet v6.0 and the D subgenomes of CS was similar to that within the segment. The difference in segment orientation between the 2 genomes was likely an assembly error.

These examples illustrate the utility of inferences on putative orthologous relationships for the detection of potential errors in the orientation and location of scaffolds in a genome assembly. This validation would be even more useful if pseudomolecules in one of the compared assemblies were nearly free of major structural errors. The hybrid approach to genome sequence assembly employing the third-generation PacBio HiFi sequencing technology and DLE-1 optical maps produces structurally robust pseudomolecules, which could be used for validation of other assemblies.

## Supplementary Material

jkaf042_Supplementary_Data

## Data Availability

The Aet v6.0 assembly is available at NCBI under Bioproject PRJNA341983. Gene annotations and optical maps are available at https://doi.org/10.5061/dryad.bvq83bkjg. Supplemental material available at G3 online.

## References

[jkaf042-B1] Camacho C, Coulouris G, Avagyan V, Ma N, Papadopoulos J, Bealer K, Madden TL. 2009. BLAST+: architecture and applications. BMC Bioinformatics. 10(1):421. doi:10.1186/1471-2105-10-421. 20003500 PMC2803857

[jkaf042-B2] Cavalet-Giorsa E, González-Munoz A, Athiyannan N, Holden S, Salhi A, Gardener C, Quiroz-Chávez J, Rustamova SM, Elkot AF, Patpour M, et al 2024. Origin and evolution of the bread wheat D genome. Nature. 633(8031):848–855. doi:10.1038/s41586-024-07808-z. 39143210 PMC11424481

[jkaf042-B3] Cheng H, Concepcion GT, Feng X, Zhang H, Li H. 2021. Haplotype-resolved de novo assembly using phased assembly graphs with hifiasm. Nat Methods. 18(2):170–175. doi:10.1038/s41592-020-01056-5. 33526886 PMC7961889

[jkaf042-B4] Cox TS, Raupp WJ, Gill BS. 1994. Leaf rust-resistance genes Lr41, Lr42, and Lr43 transferred from *Triticum tauschii* to common wheat. Crop Sci. 34(2):339–343. doi:10.2135/cropsci1994.0011183X003400020005x.

[jkaf042-B5] Cox TS, Wu J, Wang S, Cai J, Zhong Q, Fu B. 2017. Comparing two approaches for introgression of germplasm from *Aegilops tauschii* into common wheat. Crop J. 5(5):355–362. doi:10.1016/j.cj.2017.05.006.

[jkaf042-B6] Dai X, Wang H, Zhou H, Wang L, Dvorak J, Bennetzen JL, Müller H-G. 2018. Birth and death of LTR-retrotransposons in *Aegilops tauschii*. Genetics. 210(3):1053–1073. doi:10.1534/genetics.118.301198. 30158124 PMC6218219

[jkaf042-B7] Das MK, Bai G, Mujeeb-Kazi A, Rajaram S. 2016. Genetic diversity among synthetic hexaploid wheat accessions (*Triticum aestivum*) with resistance to several fungal diseases. Genet Resour Crop Evol. 63(8):1285–1296. doi:10.1007/s10722-015-0312-9.

[jkaf042-B8] Dubcovsky J, Dvorak J. 2007. Genome plasticity a key factor in the success of polyploid wheat under domestication. Science. 316(5833):1862–1866. doi:10.1126/science.1143986. 17600208 PMC4737438

[jkaf042-B9] Dvorak J, McGuire PE, Cassidy B. 1988. Apparent sources of the A genomes of wheats inferred from the polymorphism in abundance and restriction fragment length of repeated nucleotide sequences. Genome. 30(5):680–689. doi:10.1139/g88-115.

[jkaf042-B10] Dvorak J, Wang L, Zhu T, Jorgensen CM, Deal KR, Dai X, Dawson MW, Müller H-G, Luo M-C, Ramasamy RK, et al 2018. Structural variation and rates of genome evolution in the grass family seen through comparison of sequences of genomes greatly differing in size. Plant J. 95(3):487–503. doi:10.1111/tpj.13964. 29770515

[jkaf042-B11] Gaurav K, Arora S, Silva P, Sanchez-Martin J, Horsnell R, Gao L, Brar GS, Widrig V, John Raupp W, Singh N, et al 2021. Population genomic analysis of *Aegilops tauschii* identifies targets for bread wheat improvement. Nat Biotechnol. 40(3):422–431. doi:10.1038/s41587-021-01058-4. 34725503 PMC8926922

[jkaf042-B12] Gill BS, Raupp WJ, Sharma HC, Browder LE, Hatchett JH, Harvey TL, Moseman JG, Waines JG. 1986. Resistance in *Aegilops squarrosa* to wheat leaf rust, wheat powdery mildew, greenbug, and Hessian fly. Plant Dis. 70(6):553–556. doi:10.1094/PD-70-553.

[jkaf042-B13] Hastie AR, Dong L, Smith A, Finklestein J, Lam ET, Huo N, Cao H, Kwok P-Y, Deal KR, Dvorak J, et al 2013. Rapid genome mapping in nanochannel arrays for highly complete and accurate de novo sequence assembly of the complex *Aegilops tauschii* genome. PLoS One. 8(2):e55864. doi:10.1371/journal.pone.0055864. 23405223 PMC3566107

[jkaf042-B14] International Wheat Genome Sequencing Consortium (IWGSC) . 2014. A chromosome-based draft sequence of the hexaploid bread wheat (*Triticum aestivum*) genome. Science. 345(6194):1251788. doi:10.1126/science.1251788. 25035500

[jkaf042-B15] Kihara H . 1944. Discovery of the DD-analyser, one of the ancestors of *Triticum vulgare* (Japanese). Agric Hortic (Tokyo). 19:13–14.

[jkaf042-B16] Kou H, Zhang Z, Yang Y, Wei C, Xu L, Zhang G. 2023. Advances in the mining of disease resistance genes from *Aegilops tauschii* and the utilization in wheat. Plants (Basel). 12(4):880. doi:10.3390/plants12040880. 36840228 PMC9966637

[jkaf042-B17] Kuzniar A, van Ham RC, Pongor S, Leunissen JA. 2008. The quest for orthologs: finding the corresponding gene across genomes. Trends Genet. 24(11):539–551. doi:10.1016/j.tig.2008.08.009. 18819722

[jkaf042-B18] Li J, Wan HS, Yang WY. 2014. Synthetic hexaploid wheat enhances variation and adaptive evolution of bread wheat in breeding processes. J Syst Evol. 52(6):735–742. doi:10.1111/jse.12110.

[jkaf042-B19] Luo MC, Deal KR, Akhunov ED, Akhunova AR, Anderson OD, Anderson JA, Blake N, Clegg MT, Coleman-Derr D, Conley EJ, et al 2009. Genome comparisons reveal a dominant mechanism of chromosome number reduction in grasses and accelerated genome evolution in Triticeae. Proc Natl Acad Sci U S A. 106(37):15780–15785. doi:10.1073/pnas.0908195106. 19717446 PMC2747195

[jkaf042-B20] Luo M-C, Gu YQ, Puiu D, Wang H, Twardziok SO, Deal KR, Huo N, Zhu T, Wang L, Wang Y, et al 2017. Genome sequence of the progenitor of the wheat D genome *Aegilops tauschii*. Nature. 551(7681):498–502. doi:10.1038/nature24486. 29143815 PMC7416625

[jkaf042-B21] Luo M-C, Gu YQ, You FM, Deal KR, Ma Y, Hu Y, Huo N, Wang Y, Wang J, Chen S, et al 2013. A 4-gigabase physical map unlocks the structure and evolution of the complex genome of *Aegilops tauschii*, the wheat D-genome progenitor. Proc Natl Acad Sci U S A. 110(19):7940–7945. doi:10.1073/pnas.1219082110. 23610408 PMC3651469

[jkaf042-B22] Manni M, Berkeley MR, Seppey M, Simao FA, Zdobnov EM. 2021. BUSCO update: novel and streamlined workflows along with broader and deeper phylogenetic coverage for scoring of eukaryotic, prokaryotic, and viral genomes. Mol Biol Evol. 38(10):4647–4654. doi:10.1093/molbev/msab199. 34320186 PMC8476166

[jkaf042-B23] Marcais G, Delcher AL, Phillippy AM, Coston R, Salzberg SL, Zimin A. 2018. MUMmer4: a fast and versatile genome alignment system. PLoS Comput Biol. 14(1):e1005944. doi:10.1371/journal.pcbi.1005944. 29373581 PMC5802927

[jkaf042-B24] McFadden ES, Sears ER. 1946. The origin of *Triticum spelta* and its free-threshing hexaploid relatives. J Hered. 37:81–107. doi:10.1093/oxfordjournals.jhered.a105590. 20985728

[jkaf042-B25] Mistry J, Chuguransky S, Williams L, Qureshi M, Salazar GA, Sonnhammer ELL, Tosatto SCE, Paladin L, Raj S, Richardson LJ, et al 2021. Pfam: the protein families database in 2021. Nucleic Acids Res. 49(D1):D412–D419. doi:10.1093/nar/gkaa913. 33125078 PMC7779014

[jkaf042-B26] Ogbonnaya FC, Abdalla O, Mujeeb-Kazi A, Kazi AG, Xu SS, Gosman N, Lagudah ES, Bonnett D, Sorrells ME, Tsujimoto H. 2013. Synthetic hexaploids: harnessing species of the primary gene pool for wheat improvement. Plant Breed Rev. 37:35–122. doi:10.1002/9781118497869.ch2.

[jkaf042-B27] Periyannan S, Moore J, Ayliffe M, Bansal U, Wang X, Huang L, Deal K, Luo M, Kong X, Bariana H, et al 2013. The gene Sr33, an ortholog of barley *Mla* genes, encodes resistance to wheat stem rust race Ug99. Science. 341(6147):786–788. doi:10.1126/science.1239028. 23811228

[jkaf042-B28] Pertea G, Pertea M. 2020. GFF utilities: GffRead and GffCompare. F1000Res. 9:ISCB Comm J-304. doi:10.12688/f1000research.23297.2. PMC7222033 32489650

[jkaf042-B29] Raupp WJ, Amri A, Hatchettg JH, Gill BS, Wilson DL, Cox TS. 1993. Chromosomal location of hessian fly-resistance genes *H22 H23* and *H24* derived from *Triticum tauschii* in the D genome of wheat. J Hered. 84(2):142–145. doi:10.1093/oxfordjournals.jhered.a111300.

[jkaf042-B30] Rosyara U, Kishii M, Payne T, Sansaloni CP, Singh RP, Braun H-J, Dreisigacker S. 2019. Genetic contribution of synthetic hexaploid wheat to CIMMYT's spring bread wheat breeding germplasm. Sci Rep. 9(1):12355. doi:10.1038/s41598-019-47936-5. 31451719 PMC6710277

[jkaf042-B31] Shumate A, Salzberg SL. 2021. Liftoff: accurate mapping of gene annotations. Bioinformatics. 37(12):1639–1643. doi:10.1093/bioinformatics/btaa1016. 33320174 PMC8289374

[jkaf042-B32] Sun S, Wang J, Yu J, Meng F, Xia R, Wang L, Wang Z, Ge W, Liu X, Li Y, et al 2017. Alignment of common wheat and other grass genomes establishes a comparative genomics research platform. Front Plant Sci. 8:1480. doi:10.3389/fpls.2017.01480. 28912789 PMC5582351

[jkaf042-B33] Van Slageren M . 1994. Wild wheats: a monograph of Aegilops L. and Amblyopyrum (Jaub. & Spach) Eig (Poaceae). Wageningen Agricultural University.

[jkaf042-B34] Varabyou A, Sommer MJ, Erdogdu B, Shinder I, Minkin I, Chao K-H, Park S, Heinz J, Pockrandt C, Shumate A, et al 2023. CHESS 3: an improved, comprehensive catalog of human genes and transcripts based on large-scale expression data, phylogenetic analysis, and protein structure. Genome Biol. 24(1):249. doi:10.1186/s13059-023-03088-4. 37904256 PMC10614308

[jkaf042-B35] Wan H, Yang F, Li J, Wang Q, Liu Z, Tang Y, Yang W. 2023. Genetic improvement and application practices of synthetic hexaploid wheat. Genes (Basel). 14(2):283. doi:10.3390/genes14020283. 36833210 PMC9956247

[jkaf042-B36] Wang J, Luo M-C, Chen Z, You FM, Wei Y, Zheng Y, Dvorak J. 2013. *Aegilops tauschii* single nucleotide polymorphisms shed light on the origins of wheat D-genome genetic diversity and pinpoint the geographic origin of hexaploid wheat. New Phytol. 198(3):925–937. doi:10.1111/nph.12164. 23374069

[jkaf042-B37] Wang L, Zhu T, Rodriguez JC, Deal KR, Dubcovsky J, McGuire PE, Lux T, Spannagl M, Mayer KFX, Baldrich P, et al 2021. *Aegilops tauschii* genome assembly Aet v5.0 features greater sequence contiguity and improved annotation. G3 (Bethesda). 11(12):jkab325. doi:10.1093/g3journal/jkab325. 34515796 PMC8664484

[jkaf042-B38] Yang W, Liu D, Li J, Zhang L, Wei H, Hu X, Zheng Y, He Z, Zou Y. 2009. Synthetic hexaploid wheat and its utilization for wheat genetic improvement in China. J Genet Genom. 36(9):539–546. doi:10.1016/S1673-8527(08)60145-9. 19782955

[jkaf042-B39] Zhang T, Xing W, Wang A, Zhang N, Jia L, Ma S, Xia Q. 2023. Comparison of long-read methods for sequencing and assembly of Lepidopteran pest genomes. Int J Mol Sci. 24(1):649. doi:10.3390/ijms24010649. PMC9820851 36614092

[jkaf042-B40] Zhao G, Zou C, Li K, Wang K, Li T, Gao L, Zhang X, Wang H, Yang Z, Liu X, et al 2017. The *Aegilops tauschii* genome reveals multiple impacts of transposons. Nat Plants. 3(12):946–955. doi:10.1038/s41477-017-0067-8. 29158546

[jkaf042-B41] Zhu T, Wang L, Rimbert H, Rodriguez JC, Deal KR, De Oliveira R, Choulet F, Keeble-Gagnère G, Tibbits J, Rogers J, et al 2021. Optical maps refine the bread wheat *Triticum aestivum* cv. Chinese Spring genome assembly. Plant J. 107(1):303–314. doi:10.1111/tpj.15289. 33893684 PMC8360199

[jkaf042-B42] Zimin AV, Puiu D, Luo M-C, Zhu T, Koren S, Marçais G, Yorke JA, Dvořák J, Salzberg SL. 2017. Hybrid assembly of the large and highly repetitive genome of *Aegilops tauschii*, a progenitor of bread wheat, with the MaSuRCA mega-reads algorithm. Genome Res. 27(5):787–792. doi:10.1101/gr.213405.116. 28130360 PMC5411773

